# Skeletal Muscle Physiology

**DOI:** 10.1155/2013/782352

**Published:** 2013-06-06

**Authors:** Lucas Guimarães-Ferreira, Humberto Nicastro, Jacob Wilson, Nelo Eidy Zanchi

**Affiliations:** ^1^Exercise Metabolism Research Group, Center of Physical Education and Sports, Federal University of Espirito Santo, 29075810 Vitória, ES, Brazil; ^2^Laboratory of Applied Nutrition and Metabolism, School of Sports and Physical Education, University of São Paulo, 05508-030 São Paulo, SP, Brazil; ^3^Human Performance and Sports Nutrition Laboratory, The University of Tampa, Tampa, FL 33606, USA; ^4^Department of Physiology and Biophysics, University of São Paulo, 05508-900 São Paulo, SP, Brazil

In the beginning of the last century, muscle proteins were viewed as static structural molecules not capable of being utilized by other tissues or organs. This concept was accepted until the 30s, where Rudolf Schoenheimer presented strong evidences about the “Dynamic State of Body Constituents,” which means that skeletal muscle is not only capable of contracting but also capable of releasing nitrogen derived molecules to be utilized by other organs and tissues (Guggenheim, 1991) [1]. Such concept established that skeletal muscle is a highly plastic tissue, adapting its structure and metabolism in response to diverse conditions such as contractile activity, mechanical overload, and nutrients. From this point of view several questions arise, specifically, how form follows function of skeletal muscles, as well as the synergistic role of nutrients. A large number of research groups around the world are helping to clarify this and many other questions. In particular in the last four decades, the growth in the number of publications on skeletal muscle subject is noteworthy (Figure 1). 

In this special issue, the reader will be brought directly to a wide spectrum of articles regarding the skeletal muscle tissue. From France, a new hypothesis concerning how to consume amino acids and deal with catabolic conditions also is put in debate, focusing on the anabolic threshold concept (D. Dardevet et al., 2012). From the University of Tokyo, Japan, A. Wagatsuma and K. Sakuma (2013) summarize the current knowledge about the role of mitochondria as a regulator of myogenesis. From Brazil, C. O. Assumpção et al. (2013) present an extensive review on the effects of exercise-induced muscle damage on running economy in humans. Also, W. S. Dantas et al. (2013) discuss the impact of polycystic ovary syndrome on skeletal muscle tissue. A. Carsana (2013), from Italy, reviewed the documented cases of exertional rhabdomyolysis or stress-induced malignant hyperthermia and reported a possible association with RYR1 gene polymorphism. This special issue also presents original articles focusing on the cytokine response of skeletal muscle cells according its differentiation stage (M. Podbregar et al., 2013), the effects of tissue culture conditions on in vitro myogenesis (S. Hinds et al., 2013) and the differences in spontaneous physical activity between mice substrains (D. Coletti et al., 2013). All these discussions are being provided in order to generate new benefits and from athletes to debilitated populations; from basic to applied sciences. In this issue, our focus was not to restrict but, on the contrary, to be capable of proposing new hypotheses and ideas based on the current knowledge.

## Figures and Tables

**Figure 1:**
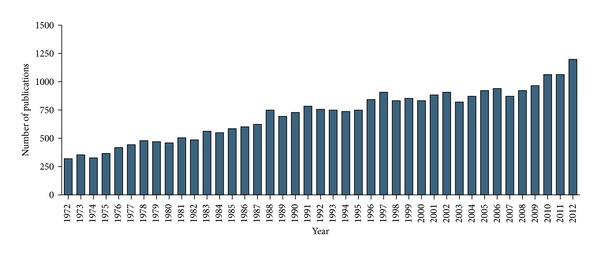
Number of publications in Medline database with “skeletal muscle” present on title. Data from the US National Library of Medicine National Institutes of Health (http://www.ncbi.nlm.nih.gov/pubmed?term=skeletal%20muscle  [Title]).
